# Supplementary material for: Sleep Benefits Memory for Semantic Category Structure While Preserving Exemplar-Specific Information
Source: Sci Rep. 2017 Nov 1;7:14869. doi: 10.1038/s41598-017-12884-5 (PMC5665979; doi:10.1038/s41598-017-12884-5)
Supplement: Supplementary file 1 — Supplementary Material [file 41598_2017_12884_MOESM1_ESM.pdf]

Supplementary material for:

Sleep Benefits Memory for Semantic Category Structure While Preserving Exemplar-Specific Information

Anna C. Schapiro, Elizabeth A. McDevitt, Lang Chen, Kenneth A. Norman, Sara C. Mednick, & Timothy T. Rogers

---

Contents of data files:

TestDataExperiment1.csv

Data from first and second test session for Experiment 1.

group:	sleep or wake
subject:	subject ID
test:	first or second test
trial:	trial number
item:	identifier for each satellite
freq:	item belonging to category exposed with low, medium, or high frequency
itemtype:	feature queried is unique, shared, or from novel satellite
subtype:	feature queried is verbal (name) or visual (part)
feature:	specific queried feature (v1-v5 refer to the five visual parts)
correct:	whether feature filled in correctly

TestDataExperiment2.csv

Data from first and second test session for Experiment 2.

Same as above except:

group:	NREM (1 minute or less of REM), REM (more than 1 minute of REM), AW (active wake), or QW (quiet wake)
stage1:	minutes of stage 1 sleep
stage2:	minutes of stage 2 sleep
stage3:	minutes of stage 3 sleep (SWS)
stageREM:	minutes of REM sleep

For training data or other information, email aschapir@bidmc.harvard.edu.

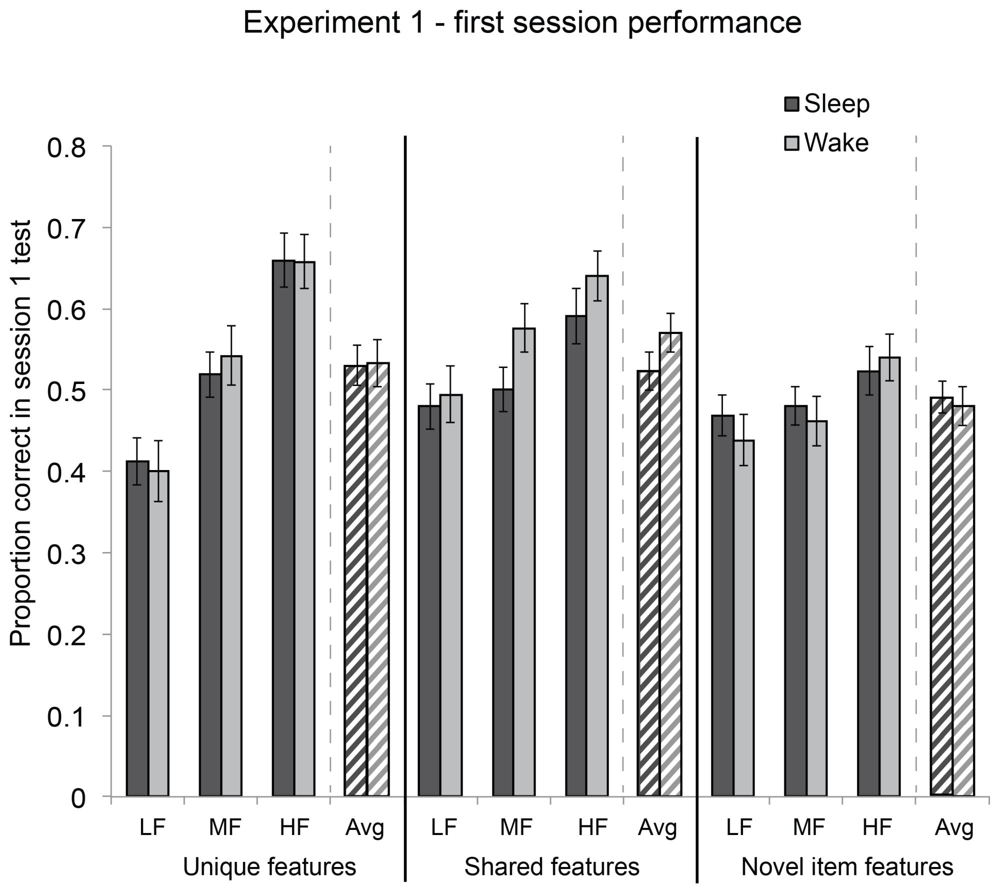

Supplementary Figure S1. Session 1 results for Experiment 1. Proportion correct in first test session for unique features, shared features, and novel item features. For each feature type, results are shown for low frequency (LF), medium frequency (MF), and high frequency (HF) category, as well as the average (Avg) across categories. Error bars denote ± 1 SEM.

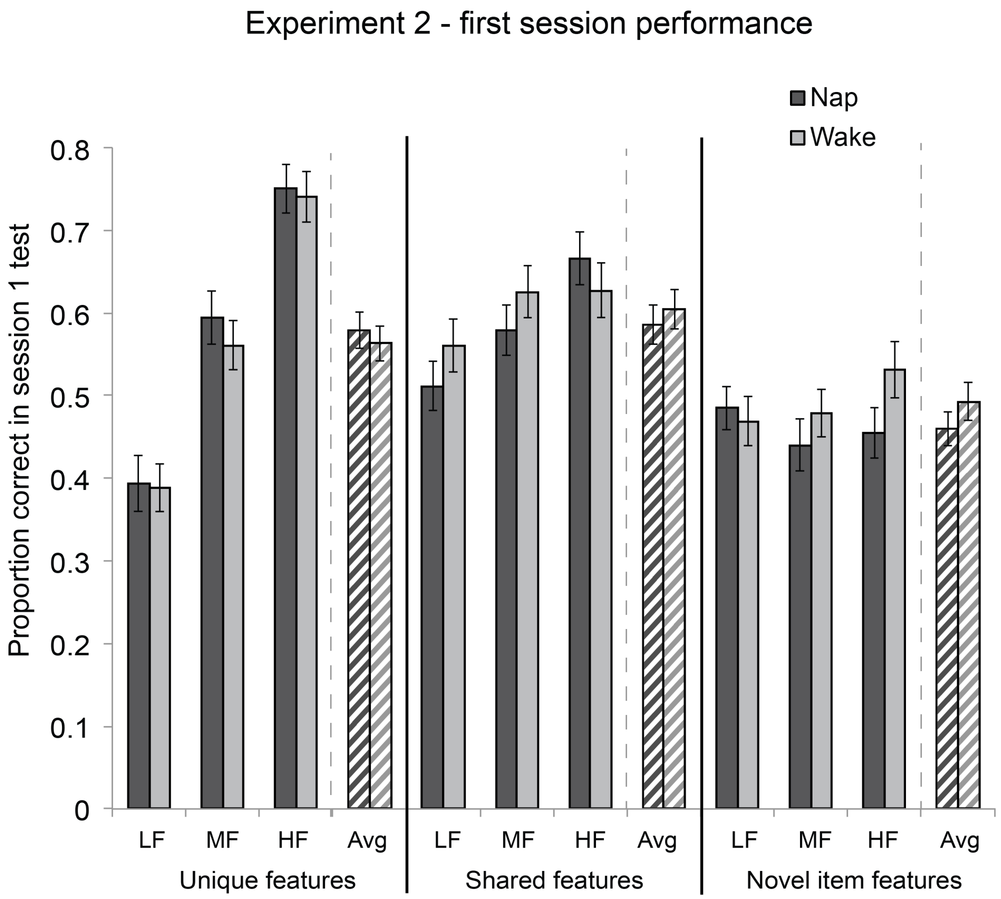

Supplementary Figure S2. Session 1 results for Experiment 2. Proportion correct in first test session for unique features, shared features, and novel item features. For each feature type, results are shown for low frequency (LF), medium frequency (MF), and high frequency (HF) category, as well as the average (Avg) across categories. Error bars denote ± 1 SEM.
